# Effects of Congenital Blindness on Ultrasonic Vocalizations and Social Behaviors in the ZRDBA Mouse

**DOI:** 10.3389/fnbeh.2022.884688

**Published:** 2022-05-03

**Authors:** Nouhaila Bouguiyoud, Elena Morales-Grahl, Gilles Bronchti, Johannes Frasnelli, Florence I. Roullet, Syrina Al Aïn

**Affiliations:** ^1^Department of Anatomy, Université du Québec à Trois-Rivières, Trois-Rivières, QC, Canada; ^2^Cognition, Neurosciences, Affect et Comportement (CogNAC) Research Group, Université du Québec à Trois-Rivières, Trois-Rivières, QC, Canada; ^3^Carleton College, Northfield, MN, United States; ^4^Department of Psychiatry and Behavioural Neurosciences, McMaster University, Hamilton, ON, Canada

**Keywords:** congenital blindness, acoustic communication, social behavior, mice, development

## Abstract

Mice produce ultrasonic vocalizations (USVs) at different ages and social contexts, including maternal-pup separation, social play in juveniles, social interactions, and mating in adults. The USVs' recording can be used as an index of sensory detection, internal state, and social motivation. While sensory deprivation may alter USVs' emission and some social behaviors in deaf and anosmic rodents, little is known about the effects of visual deprivation in rodents. This longitudinal study aimed to assess acoustic communication and social behaviors using a mouse model of congenital blindness. Anophthalmic and sighted mice were assayed to a series of behavioral tests at three different ages, namely, the maternal isolation-induced pup USV test and the home odor discrimination and preference test on postnatal day (PND) 7, the juvenile social test on PND 30–35, and the female urine-induced USVs and scent-marking behavior at 2–3 months. Our results evidenced that (1) at PND 7, USVs' total number between both groups was similar, all mice vocalized less during the second isolation period than the first period, and both phenotypes showed similar discrimination and preference, favoring exploration of the home bedding odor; (2) at PND 30–35, anophthalmic mice engaged less in social behaviors in the juvenile play test than sighted ones, but the number of total USVs produced is not affected; and (3) at adulthood, when exposed to a female urine spot, anophthalmic male mice displayed faster responses in terms of USVs' emission and sniffing behavior, associated with a longer time spent exploring the female urinary odor. Interestingly, acoustic behavior in the pups and adults was correlated in sighted mice only. Together, our study reveals that congenital visual deprivation had no effect on the number of USVs emitted in the pups and juveniles, but affected the USVs' emission in the adult male and impacted the social behavior in juvenile and adult mice.

## Introduction

In the animal kingdom, most of the species emit vocalizations in response to various social stimuli. House mice are known to produce mainly ultrasonic vocalizations (USVs), characterized by fundamental frequency spanning a range of 35–110 kHz (Sales, 1972; Branchi et al., 1998; Holy and Guo, 2005). A vast number of studies have documented that the USVs' features may be modulated as a function of social contexts and the developmental stage of the mouse emitter (Nyby, 1983; Maggio and Whitney, 1985; Ehret, 2005; Holy and Guo, 2005; Wang et al., 2008; Williams et al., 2008; Grimsley et al., 2011; Hanson and Hurley, 2012; Ey et al., 2013; Sangiamo et al., 2020). Specifically, vocalization behavior was first studied in rodent pups during the isolation of the pups from their mothers and littermates, resulting in USVs' calls between birth and postnatal day (PND) 14 with a peak emission at the age of 7–9 days (Sales, 1972; Branchi et al., 1998; Ehret, 2005; Fischer and Hammerschmidt, 2011). USVs' emission has been reported in juvenile mice during social interactions/play with an age-/sex-matched congener (Panksepp et al., 2007), just like in both male and female adult mice during dyadic encounters, courtship, and mating (Pomerantz et al., 1983; Maggio and Whitney, 1985; Holy and Guo, 2005; Hammerschmidt et al., 2009; Scattoni et al., 2009; Grimsley et al., 2011; Roullet et al., 2011; Wöhr and Schwarting, 2013; von Merten et al., 2014). The acoustic behavior is also regulated by internal state, such as the strength of arousal and emotion (Brudzynski, 2013; Gaub et al., 2016; Grimsley et al., 2016; Boulanger-Bertolus et al., 2017; Demir et al., 2020), and by external factors/conditions, such as the presence of a predator/attractive congener (Sales, 1972; Mun et al., 2015). Furthermore, social behavior and communication are tightly linked as demonstrated by other studies focusing on acoustic communication and aberrant social interactions in models of neurodevelopmental disorders, such as mouse models of autism spectrum disorder or fragile X syndrome (Jamain et al., 2008; Scattoni et al., 2008; Bozdagi et al., 2010; Wöhr et al., 2011; Schmeisser et al., 2012; Ey et al., 2013; Wöhr, 2014; Belagodu et al., 2016). Taken together, quantifying USVs' emission in diverse social contexts helps in assessing the dynamics underlying socioaffective communication in rodent models, and is thus relevant to better interpret alterations in vocal communication and sociability seen in rodent models of disorders (Wöhr and Scattoni, 2013).

Importantly, USVs' emission may rely on the integrity of sensory systems as sensory—mainly auditory and olfactory—disruption leads to substantial changes in USVs' features. Indeed, early deafened mice emitted intact USVs' rates in pup isolation and male's courtship contexts (Hammerschmidt et al., 2012; Mahrt et al., 2013), whereas late deafened male mice resulted in increased female urine-induced social vocalizations (Arriaga et al., 2012). Moreover, disruption of the vomeronasal system led to considerable reduction of USVs' levels emitted by males in response to female stimulus, although ZnSO_4_-induced anosmia did not alter the USV numbers (Bean, 1982). Notwithstanding, the importance of the visual inputs on acoustic communication and related social behaviors has received little attention so far. Interestingly, Langford et al. (2006) highlighted that, in mice, visual cues are required to trigger empathic responses toward a congener in pain as an opaque screen abolished empathic responses, whereas deaf and anosmia did not. Nevertheless, congenitally blind women displayed increased vocalizations toward their newborn, accompanied by heightened duration of contact/proximity and breastfeeding compared with sighted dyads (Thoueille et al., 2006; Chiesa et al., 2015; Ganea et al., 2018). To the best of our knowledge, the link between visual inputs, acoustic communication, and related social behaviors has not been examined in rodent models of visual deprivation.

This study aimed to investigate USVs and social behaviors across development in a congenitally blind mouse model, called ZRDBA strain. Both anophthalmic and sighted phenotypes were assayed to four behavioral tests, namely, (1) the maternal isolation-induced pup USV test consists in recording USVs numbers emitted by PND 7 pups for twice 5-min periods of maternal isolation; (2) the home odor discrimination and preference test consists in measuring USVs' levels associated to time spent exploring the home and clean bedding odors in PND 7 pups; (3) the juvenile social test consists in quantifying USVs' calls, social and nonsocial behaviors of an experimental mouse exposed to a non-familiar congener (sex and age matched); and (4) the female urine-induced USVs and scent-marking behavior test consists in exposing adult males to female urine and recording their USVs emission and sniffing/markings behaviors.

The ZRDBA strain has been obtained by crossbreeding between the anophthalmic ZRDCT and the sighted DBA-6 strains (Touj et al., 2019). The anophthalmic ZRDCT mice have orbits but neither eyes, nor optic tracts and afferents retina-hypothalamus due to a mutation on chromosome 18 of the Rx/Rax gene (Chase and Chase, 1941). The crossbreeding results in a litter with an equal number of anophthalmic Rx/Rax homozygous and sighted Rx/Rax heterozygous pups. Interestingly, a deformation-based morphometry study conducted on ZRDBA adult mice highlighted structural alterations of the ventromedial hypothalamus, the preoptic area, and the bed nucleus of stria terminalis (Touj et al., 2020, 2021); these cerebral regions being implicated in the mediation of the social communication and social behaviors, such as aggression, mating, parental care, and defense (Lebow and Chen, 2016). In the light of these neuroimaging data, we hypothesized that anophthalmic mice might show deficits in social behavior associated with lower numbers of USVs compared with sighted mice (once eyes opening).

## Methods

### Animals

In this study, we used 26 anophthalmic mice (10 females and 16 males) and 20 sighted mice (four females and 16 males) in total. All mice were bred and housed in mixed cages of 3–6 blind and sighted individuals in the animal facility. Standard and constant housing conditions were applied including a 14/10 h light/dark cycle, a free and *ad libitum* access for water/food, and a controlled room temperature set at 21°C and 40–60% humidity. Pups were tattooed on the paw for identification at PND 1. In the ZRDBA strain, breeding a blind mouse with a sighted mouse generates litters comprising half the pups born blind (homozygous, results in the absence of eyes and optic nerves), and the other half born sighted (heterozygous). All experimental procedures were approved by the animal care committee of Université du Québec à Trois-Rivières in accordance with the Canadian Council on Animal Care guidelines.

### Behavioral Procedures

**Figure 1:**
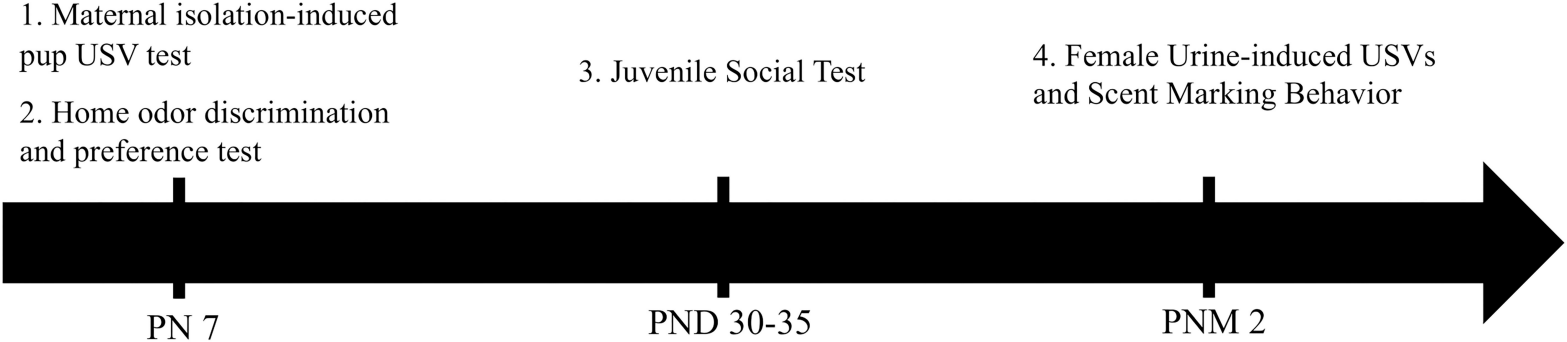
Timeline of the experimental design.

**Figure 2:**
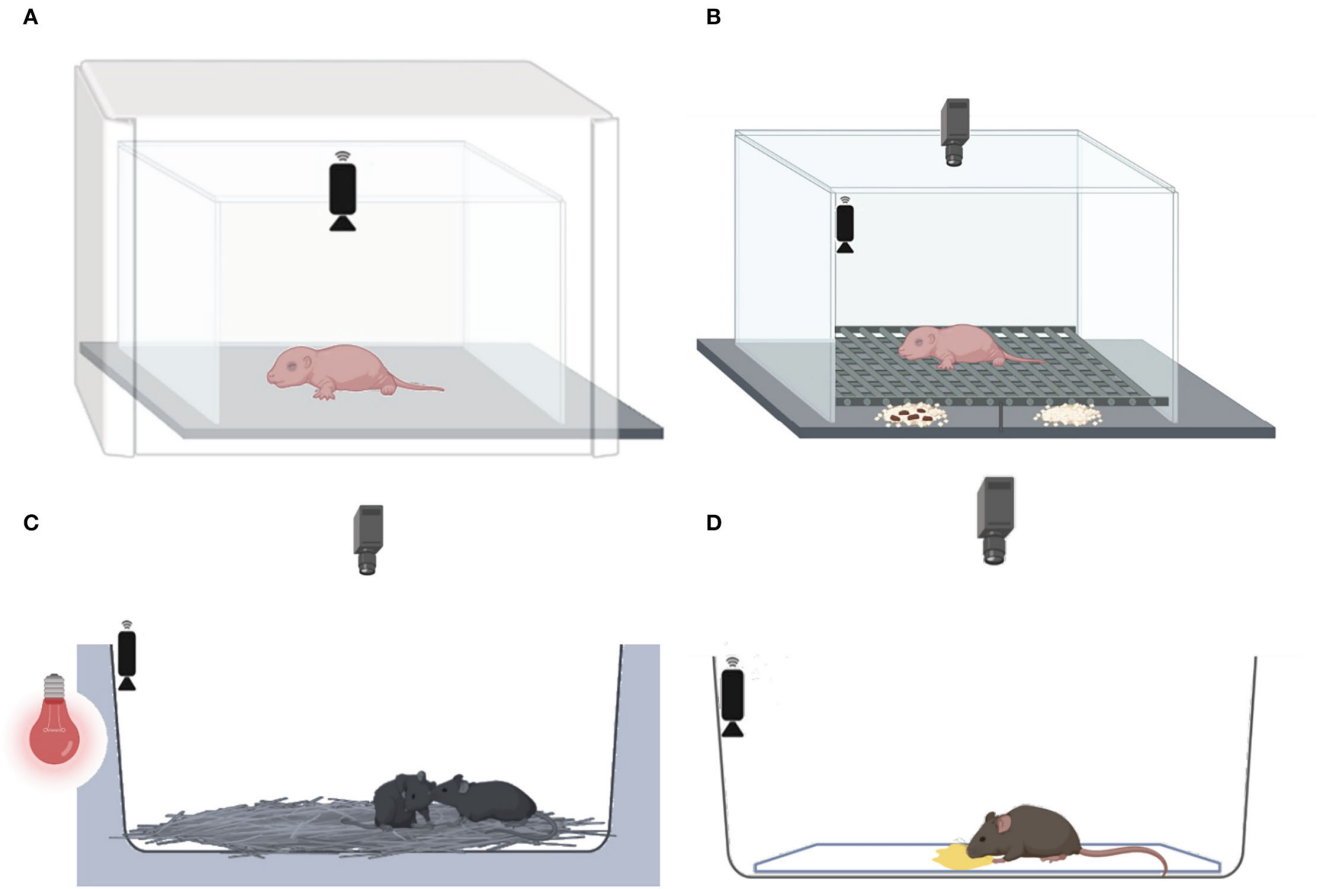
Behavioral tests conducted at three developmental stages. **(A)** At PND 7, the maternal isolation-induced pup USV test, followed by **(B)** the home odor discrimination and preference test, **(C)** at PND 30–35, the juvenile social test, and **(D)** at 2–3 months, the female urine-induced USVs and scent-marking behavior test.

Behavioral experiments carried out during the light phase between 9:00 a.m. and 5:00 p.m. All mice were assayed to the following behavioral tests conducted at three developmental stages (see Figures 1, 2), namely, (1) at PND 7, the maternal isolation-induced pup USV test, followed by the home odor discrimination and preference test; (2) at PND 30–35, the juvenile social test; and (3) at 2–3 months, the female urine-induced USVs and scent-marking behavior test. At PND 7, all the pups from all litters were assayed to the maternal isolation-induced pup USVs test and the home odor discrimination and preference test (*n* = 26 blind and 20 sighted mice). At PND 35, all of the mice from all litters were tested as experimental mice in the juvenile social test, except for mice used as social stimuli (*n* = 15 blind and 14 sighted). All males were assessed in female urine-induced USVs and scent-marking behavior (*n* = 16 blind and 16 sighted mice).

All behavioral recordings and codings were performed using Ethovision XT software (Noldus, VA, USA). To record USVs' rates, an ultrasonic microphone was suspended above or attached to the wall of the experimental cage according to the behavioral tests. USVs' recordings were processed and analyzed through the UltraVox XT system (Noldus Information Technology, The Netherlands). The system can record the full spectrum of sound and has a maximum frequency of 160 kHz.

#### Maternal Isolation-Induced Pup USV Test

The maternal isolation-induced pup USV test was adapted from Branchi et al. (2006). A 30-min habituation period was performed to acclimate the dams and their littermates to the experimental room. Dams were then isolated from their littermates and placed in an individual cage (28 × 18 × 12 cm). The testing chamber was a sound-attenuation chamber (24 × 24 × 27.5 cm) with a temperature-controlled pad set at 22°C. The testing consisted in, first, introducing a PND 7 pup within the testing chamber and recording the rate of USVs' calls produced for 5 min using an ultrasonic microphone suspended at 5 cm above the center of the testing chamber (isolation 1). Secondly, the pup was placed into the cage containing his dam for 3 min (maternal-pup reunion). Finally, the pup was reintroduced into the testing chamber, and USVs' rates were again recorded for 5 min (isolation 2).

#### Home Odor Discrimination and Preference Test

The home odor discrimination and preference test, adapted from Roullet et al. (2010) and Meyer and Alberts (2016), was conducted 5 min after the previous test. Each PND 7 pup was individually placed to the center of the testing cage (35 × 20 × 20 cm) with mesh flooring under which one side contained a cup with clean bedding and the other side contained a cup with home bedding (3 g each). The time spent exploring each cup and USVs' rates were measured for 2 min. Testing cage was cleaned with 50% ethanol, and bedding was changed between each pup testing. At the end of the test, pups were weighed, and their body temperature was noticed.

#### Juvenile Social Test

Juvenile social behavior and USVs' assessments, based on Panksepp et al. (2007) and Kabir et al. (2014), consisted in studying dyadic encounters between non-familiar sex and age-matched mice (PND 30–35). USVs in the social play test are considered as a relevant index of social motivation (Panksepp et al., 2007). The experimental mouse and social stimulus mouse were habituated to the experimental room for 1 h before the testing, during which each mouse was placed into an individual cage (28 × 18 × 12 cm), with clean bedding and without access to food and water, to improve social interest. The stimulus mice were all sighted and were previously habituated to encounter unfamiliar mice to attenuate potential stress effects. The experimental mouse was placed first into the testing cage (44.5 × 24 × 19.5 cm) for a 10–min habituation period. Once the social stimulus was introduced in the cage, social, non-social behaviors and USVs exhibited by the pairs of experimental and stimulus juvenile mice (blind-sighted and sighted-sighted) were recorded for 10 min.

The scored behaviors were adapted from Willey et al. (2009) and Cox and Rissman (2011). Social behaviors refer to (1) social sitting (sitting when being in close contact with the mouse stimulus), (2) social sniffing (investigating the mouse stimulus by sniffing the nose or the anogenital zone), and (3) following (walking behind the mouse stimulus). Non-social behaviors refer to (1) self-grooming, (2) exploring (investigating the cage alone), and (3) sitting alone. Mice were eventually weighed and put back in their home cage.

#### Female Urine-Induced USVs and Scent-Marking Behavior

This protocol, based on Roullet et al. (2011) and Binder et al. (2020), was conducted on adult male mice at 2–3 months. USVs are considered as an indicator of social motivation and sexual arousal in this social context (Wöhr and Scattoni, 2013). First, 1 week before the experimental testing, the male mouse was previously exposed to an unfamiliar female mouse, which is a crucial step to elicit subsequent male USVs' emission. Practically, both male and female mice were introduced for 5 min in a clean cage separated by a mesh to prevent copulation. Second, 24 h before testing, we placed soiled bedding of the male into the home cage of the female to induce the estrus. On the day of testing, the vaginal area was checked, and, if it was red, inflamed, and open, the female mouse was considered to be in estrus phase. A urine sample was thus performed by holding the female and gently stroking her abdomen toward the caudal direction. Fresh urine was collected in an Eppendorf tube and used for testing within 15 min.

After a 30-min habituation period in the experimental room, the male was then acclimated to the experimental cage (44.5 × 24 × 19.5 cm) for 30 min with a Strathmore paper (Strathmore Sketch Paper Pad, microperforated, 300 series, WI, USA) lining the floor and a small amount of its own bedding placed in a corner. Mice were then placed back into their own cage, while the experimental cage was cleaned (removal of the bedding) and urine markings were detected using an ultraviolet lamp and were circled with a marker. Later, 100 μl of urine from the familiar female were placed on the Strathmore paper and the test started once the male mouse was placed in the experimental cage (Lehmann et al., 2013). The total numbers of USVs, total numbers of scent markings, time spent exploring the female urine, and total distance traveled were videotaped and analyzed for 5 min. Once the test ended, the Strathmore paper was removed, Ninhydrin was sprayed (DavTech Company, Canada), and scent markings were visible after 24 h. To quantify the scent marking, a transparent grid with 1 cm^2^ squares was put on the Strathmore paper, and each square soiled by a urine marking was counted as one scent-mark unit. The total numbers of scent marks in the whole cage and within 10 cm^2^ around the female urine spot were counted (Roullet et al., 2011).

### Statistical Analysis

We used SPSS 22.0 (IBM, Armonk, NY, USA) for statistical analysis. We verified normal data distribution using the Shapiro-Wilk/Kolmogorov-Smirnov test. For data not normally distributed, we used the Mann-Whitney test to compare the experimental groups. All data are shown as mean ± SE. Regarding the maternal isolation-induced pup USV test, we computed a repeated-measures ANOVA with time (two levels—isolation 1 and isolation 2) as within-subject factor and group (two levels—anophthalmic mice and sighted mice) as between-subject factor. Regarding the home odor discrimination and preference, we computed odor preference of each mouse as a difference index: time spent above home bedding odor—clean bedding odor (Meyer and Alberts, 2016). Consequently, we compared odor preference score and USV rates between the groups with Mann-Whitney *U*-tests for independent samples. Regarding the juvenile social and the scent-marking behavior tests, we compared both groups using a one-way MANOVA (independent groups—two visual groups; dependent variables—measures within a test). We examined correlations between variables for each group using Spearman's correlation. For all analyses, we set the significance level at *p* < 0.05, with appropriate corrections for multiple comparisons (Bonferroni's correction).

## Results

Statistical analysis revealed no significant effect of sex or of the interaction sex ^*^ vision on each dependent variable (*p* > 0.05); therefore, data from both sexes were combined.

### Maternal Isolation-Induced Pup USV Test

**Figure 3:**
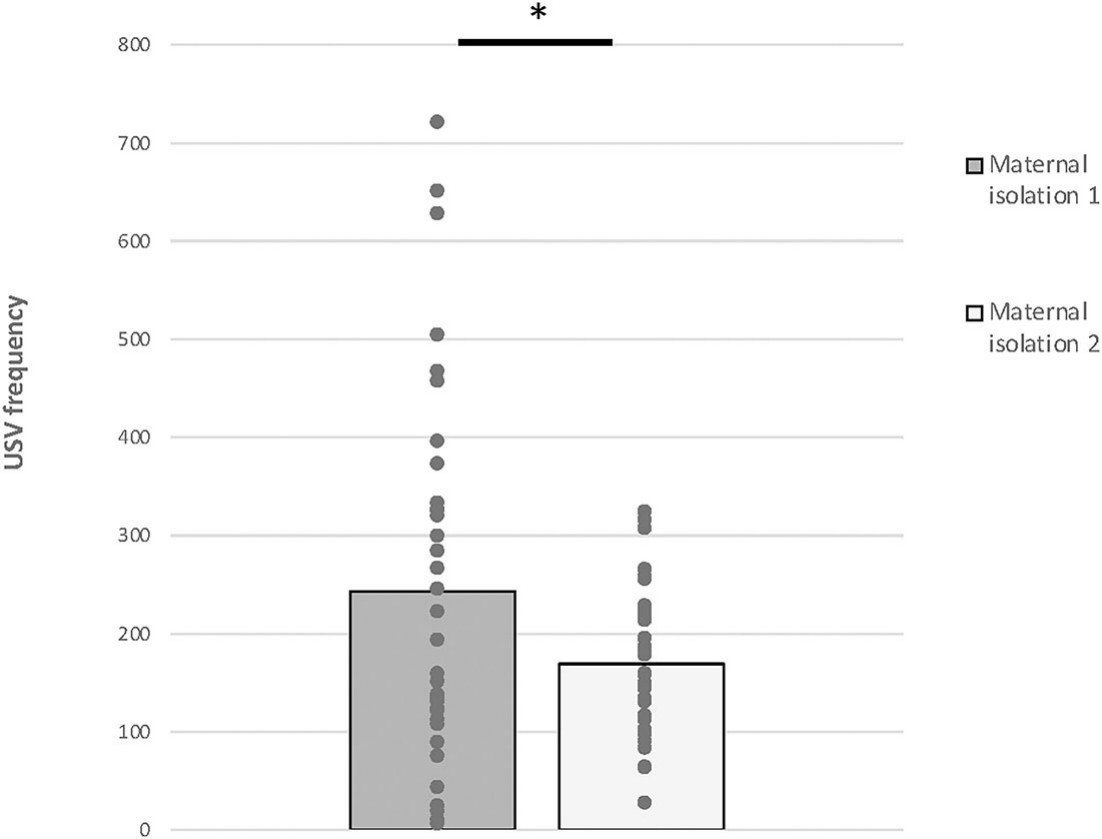
Maternal isolation-induced pup USV test. Total numbers of USVs emitted by PND 7 mice (data from anophthalmic and sighted mice were compiled) during the 5-min isolation 1 and the 5-min isolation 2 (mean ± SE; *p < 0.05).

Repeated-measures ANOVA analysis revealed a significant effect of *time* [*F*_(1,36)_ = 6.885, *p* = 0.013], but we did not find any effect of *visual status* [*F*_(1,36)_ = 0.854, *p* = 0.362] or the interaction *time* ^*^ *visual status* [*F*_(1,36)_ = 2.973; *p* = 0.094] (Figure 3).

### Home Odor Discrimination and Preference Test

**Figure 4:**
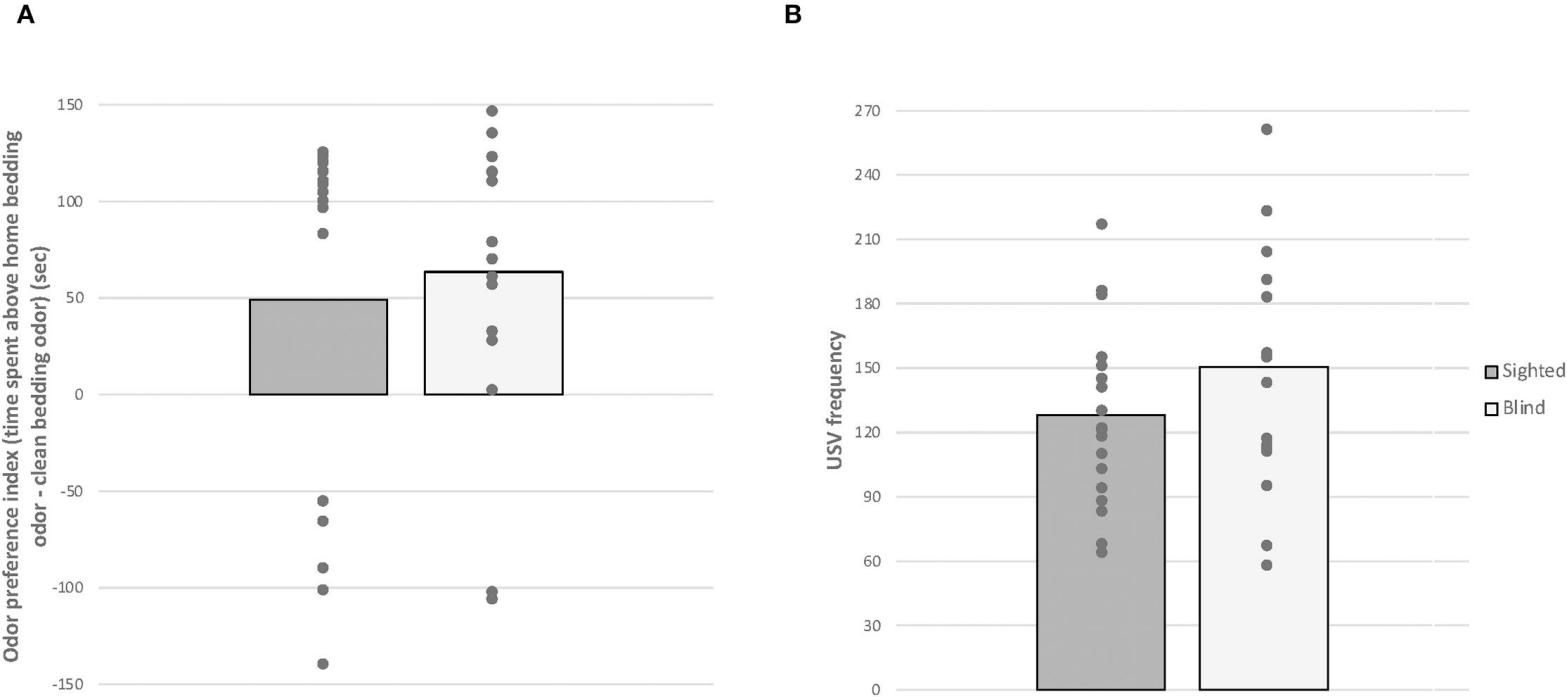
Home odor discrimination and preference test. Anophthalmic and sighted mice assayed to the home odor discrimination and preference test: **(A)** Time spent (s) exploring the home bedding odor minus the clean bedding odor and **(B)** total numbers of USVs produced by PND 7 pups during the 2-min test (mean ± SE).

Given that data for odor preference index and USV rates did not pass the Shapiro-Wilk normality test (*W* = 0.719, *p* < 0.001 and *W* = 0.852, *p* = 0.015, Figure 4), we performed Mann-Whitney tests. There was no significant effect of odor preference index and USVs' rates between both groups (*U* = 155, p = 0.888; *U* = 197.5, *p* = 0.236). In addition, no correlation was found between the number of USVs and the odor preference index in both phenotypes [blind: *r*(16) = −0.147, sighted: −0.383, *p* > 0.05].

### Juvenile Social Test

**Figure 5:**
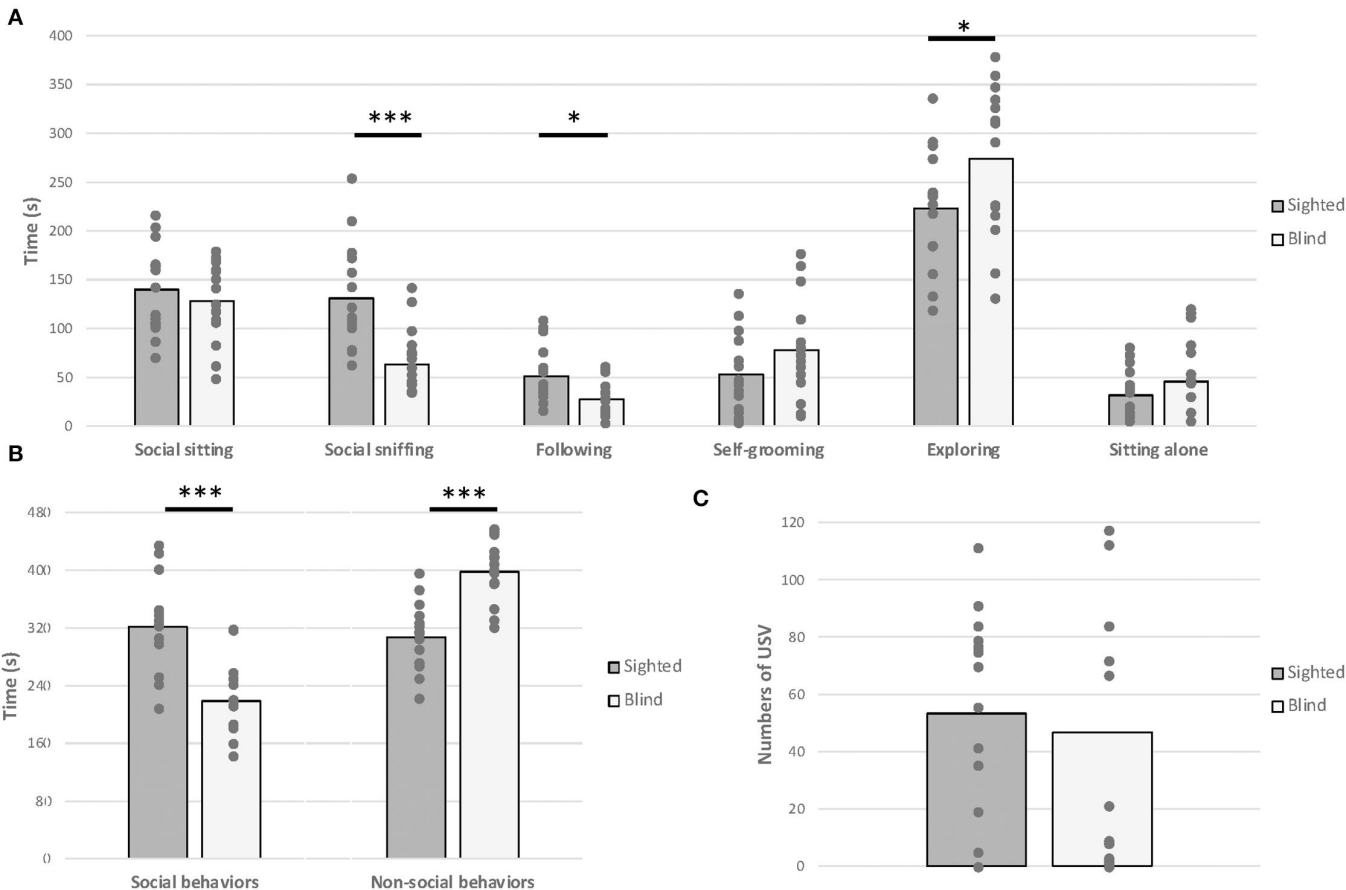
Juvenile social test. Anophthalmic and sighted mice assayed to a 10-min juvenile social test at PND 30–35: **(A)** Mean (± SE) of the time spent (s) displaying social behaviors, such as social sitting, social sniffing, following the congener, and non-social behaviors, such as exploring, sitting alone, and self-grooming. **(B)** Mean (± SE) of the time spent (s) displaying social behaviors and non-social behaviors. **(C)** Mean (± SE) of total number of USV calls produced (*p < 0.05; ***p < 0.001).

One-way MANOVA test revealed a significant effect of *visual status* on behavioral variables measured [*F*_(9,19)_ = 2.925, *p* = 0.023, Figure 5]. Specifically, blind mice exhibited less time spent in sniffing [*F*_(1,27)_ = 15.635, *p* < 0.001] and in following the congener [*F*_(1,27)_ = 6.499, *p* = 0.017] compared to sighted mice. Moreover, blind mice spent more time exploring the cage alone [*F*_(1,27)_ = 3.977, *p* = 0.046]. Overall, Student's *t-*tests confirmed that blind mice engaged less social behaviors [*t*_(1,27)_ = 4.772, *p* < 0.001] and consequently more frequent non-social behaviors [*t*_(1,27)_ = −5.419, *p* < 0.001]. Nevertheless, the total number of USVs emitted was similar between both groups [*F*_(1,27)_ = 0.202, *p* = 0.657].

In addition, we did not find any correlation between the total number of USVs and any social or non-social behaviors in both phenotypes [blind: r(16) = −0.335 to 0.253, sighted: −0.473 to 0.399, *p* > 0.05]. In blind mice, time spent exploring alone the environment was positively correlated with distance traveled [r(16) = 0.526, *p* = 0.044] and negatively associated with time spent sniffing the congener [r(16) = −0.601, *p* = 0.018], following the congener [r(16) = −0.532, *p* = 0.041], and sitting alone [r(16) = −0.561, *p* = 0.030]. In sighted mice, time spent following the congener was positively correlated with time spent sniffing this latter [r(16) = 0.572, *p* = 0.033] and negatively correlated with time spent sitting close to it [r(16) = −0.554, *p* = 0.040]. Besides, time spent exploring the cage in sighted mice was negatively associated with time spent self-grooming [r(16) = −0.688, *p* = 0.007].

### Female Urine-Induced USVs and Scent-Marking Behavior

**Figure 6:**
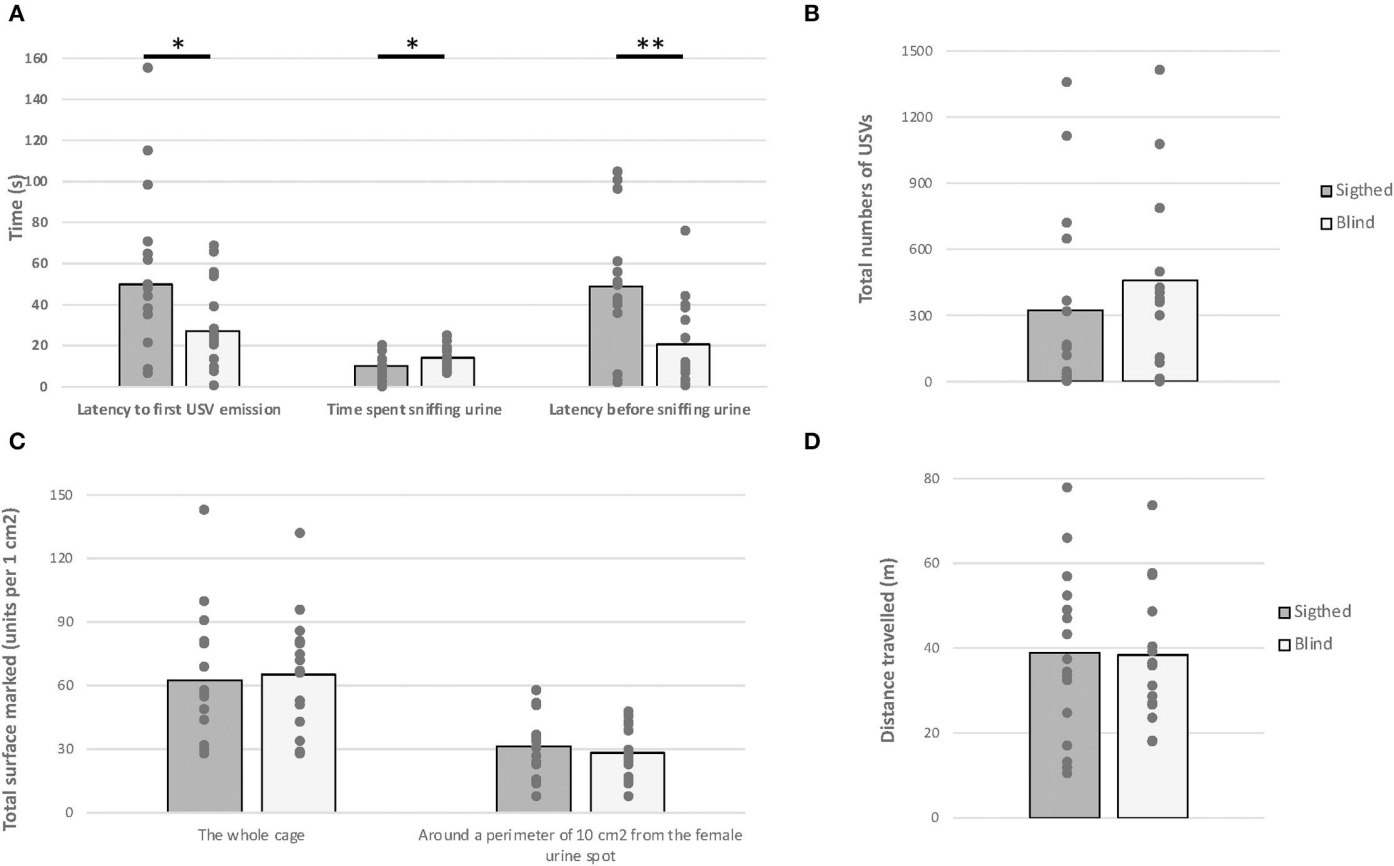
Female urine-induced USVs and scent-marking behavior. Anophthalmic and sighted male mice were exposed to a 100–ml female urine spot for 5 min: **(A)** The latency before emitting the first USV call, the time spent sniffing the female urine, the latency before sniffing the female urine; **(B)** the total numbers of USVs emitted; **(C)** the total number of mark units; and **(D)** the total distance traveled (*p < 0.05; **p < 0.01).

The one-way MANOVA revealed a significant effect of *visual status* on behavioral variables measured [*F*_(6,25)_ = 2.560, *p* = 0.040, Figure 6]. Specifically, blind mice were faster to sniff the urine spot and spent more time sniffing it [*F*_(1,30)_ = 8.934, *p* = 0.006, *F*_(1,30)_ = 4.580, *p* = 0.040, respectively]. Additionally, blind mice emitted USV faster than their sighted counterparts [*F*_(1,30)_ = 4.679, *p* = 0.039]. No difference was demonstrated in both groups in terms of total numbers of USVs, distance traveled, and total surface marked [*F*_(1,30)_ = 0.715; *F*_(1,30)_ = 0.008; *F*_(1,30)_ = 0.404, *p* > 0.05, respectively].

In addition, no correlation was found between the total number of USVs and any behavioral variables in blind mice [r(16) = −0.462 to 0.370, *p* > 0.05], whereas a positive correlation was found between the total number of USVs and (1) the total numbers of scent marks and (2) the latency before emitting USVs' calls in sighted mice [r(16) = 0.593, *p* = 0.015; r(16) = 0.535, *p* = 0.033, respectively]. Finally, the more sighted mice sniff the urine spot, the more they mark it [r(16) = 0.620, *p* = 0.010].

### Correlations Between USVs' Rates

**Figure 7:**
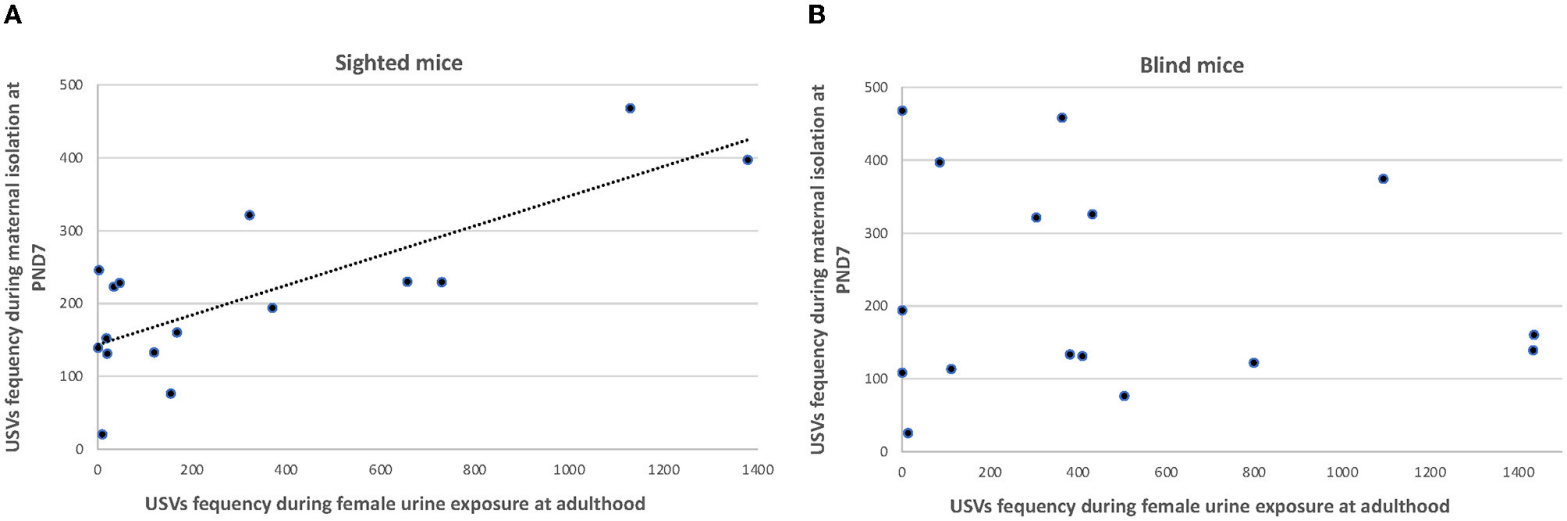
Correlation graph of the total number of USVs emitted by PND 7 pups during the first 5-min isolation and during the scent-marking test in male adults **(A)** [sighted mice: r(16) = 0.599, *p* = 0.014], and **(B)** blind mice.

In blind mice, there was no correlation between the total number of USVs calls produced in the pup isolation, odor preference, social juvenile, and scent-marking tests. In sighted mice, a positive correlation was found between the total number of USVs emitted by PND 7 pups during the first 5-min isolation and during the scent-marking test in male adults [r(16) = 0.599, *p* = 0.014, Figure 7].

## Discussion

This study assessed acoustic communication and related behaviors in a mouse model of congenital blindness using a longitudinal approach. At PND 7, both sighted and blind mice exhibited similar social behavior and USVs' calls. In contrast, at PND 35, both groups differed significantly on several social behaviors, but no difference in USVs. Specifically, juvenile blind mice exhibited spent more time exploring the cage, and spent less time following and sniffing a congener than sighted ones. In male adults in response to a female urine stimuli, blind mice displayed shorter latency to vocalize and longer time spent sniffing the female urine spot, suggesting enhanced odor acuity and/or localization induced by early blindness. Finally, correlation analyses showed that in sighted mice the number of USVs emitted during the first maternal isolation at PND 7 was positively correlated with the number of USVs emitted during scent-marking test and a positive correlation was found, in the scent-marking test, between the number of USVs produced and the total number of scent markings. Conversely, in blind mice, no significant correlations were observed.

### Early Developmental Stage (PND 7): USV Emission and Social Odors Discrimination and Preference

Isolation-induced USV has been widely employed as a marker for distress/anxiety in pups' rodents. USVs emitted by pup mice and rats are a spontaneous response to isolation from their mother (Barnes et al., 2017). Our results revealed no differences in the number of USVs emitted during the first and/or the second maternal isolation between sighted and blind pups. At PND 7, visual impairment had no incidence on isolation induced-vocalization behavior. Both groups having their eyes closed at this early developmental stage [the eyes opening in sighted mice occurs around PND 11–13 (*personal observations*)], we can assume that the visual status of both groups is similar. This indicates that the possible neuroanatomical differences between sighted and anophthalmic pups, such as a lack of optic nerve (Touj et al., 2019), have no impact on USVs' production behavior at PND 7. In this study, we also observed no difference between anophthalmic and sighted pups' calls emission during a second maternal isolation phase compared to the first phase, both groups emitting less calls in the second period. Hence, we did not observe the “maternal potentiation” phenomenon, namely, a higher acoustic response induced by a second isolation phase, which has been described previously in several studies in rats (Hofer et al., 1994; Shair, 2007) and in Swiss-Webster mice at PND 7–8 (Scattoni et al., 2008, 2009). Our results, however, are consistent with other studies, which showed no or decreasing number of USVs produced during the reisolation period in C57BL/6J mice (Barnes et al., 2017) and guinea pigs (Hennessy et al., 2006), respectively.

Besides USVs' emission, we also observed that both anophthalmic and sighted 7-day-old pups had similar olfactory behaviors. Indeed, both groups were able to discriminate and preferred exploring their home bedding's compared to the clean bedding's odors. These results are consistent with several studies showing that 9- to 13-day-old mice express their preference for their own nest to a clean one by searching and reaching the home nest/bedding area and spending more time on it (Scattoni et al., 2008; Lo Iacono et al., 2021; Premoli et al., 2021). It has also been suggested that, prior to the opening of the eyes pups' exploratory behavior is exclusively driven by olfactory cues (Freeman and Rosenblatt, 1978). Our findings indicate that both genotypes displayed the same odor performance in terms of odor discrimination and preference.

Taken together, our congenital blindness mouse model did not present alteration of neither the number of isolation-induced USVs emitted nor social olfactory behaviors at PND 7.

### Juvenile Developmental Stage: PN30-35

During social play, the pairs of blind-sighted and sighted-sighted juvenile mice displayed a similar number of USVs. Interestingly, social behavior was altered in the blind mice, with a heightened time exploring the cage to the detriment of sniffing and following the congener. No differences between sighted and anophthalmic juvenile mice were observed otherwise in self-grooming, social sitting, and sitting-alone behaviors. Consistent with previous studies (Klein and Brown, 1969; Dyer and Weldon, 1975; Iura and Udo, 2014), we recently reported that congenitally blind mice exhibited an enhanced motivational level to explore a new environment, such as seen in the open-field, Elevated Plus Maze (EPM), and Forced Swim tests, compared to sighted controls (Bouguiyoud et al., 2022). The hyperactivity observed in blind mice when exploring a novel environment may reflect a compensatory mechanism by which the lack of visual information is counterbalanced by gathering and memorizing information from the physical world before it becomes familiar (Iura and Udo, 2014). Accordingly, blind animals may require a longer habituation period to explore the new environment. This could explain their increased general exploratory behavior despite the presence of a social stimulus mouse.

Furthermore, this apparent preference for exploring the environment over engaging in social interactions with a congener resembles the social deficits described in mouse models of autism (Moy et al., 2004; McFarlane et al., 2008; Silverman et al., 2010). Animal models of autism showed social behavior deficits such as less time sniffing, grooming, and following an unfamiliar congener (Brodkin, 2007; McFarlane et al., 2008) and increased time allocated to self-grooming and inactivity (McFarlane et al., 2008).

Interestingly, in humans, a link between congenital blindness and autism was proposed based on the dramatic increased prevalence autism in individuals suffering visual impairments (Jure et al., 1991; Hobson et al., 1999; Hobson, 2011; Suhumaran et al., 2020). Moreover, children with profound visual impairment show delays in the development of joint attention behaviors, such as sharing or talking about their interests to others (Tadić et al., 2010; Dale et al., 2014). However, social behavioral differences in visually impaired individuals may be caused by specific sensory limitations, as suggested by Chokron et al. (2020), and the link between early visual deprivation, social communication, and social behavior throughout development is still unclear in humans and non-human animal models.

Taken together, the similar amount of calls in the pairs of juveniles with a lower amount of social interaction in the blind-sighted mice indicates that either (1) blind-sighted pairs of mice produced higher amounts of USVs in a shorter total duration of social interactions than the sighted-sighted ones or (2) the calls were emitted during social and non-social exploratory behaviors. It should be noted that blind animals developed ultrasonic echolocation abilities to help them explore efficiently their surroundings (Griffin, 1944; Dufour et al., 2005; Schenkman and Nilsson, 2010; Thaler et al., 2011; Kupers and Ptito, 2014). Future studies should track each animal and record their acoustic behavior emitted specifically during social behaviors, such as allogrooming, sniffing, following, or specific non-social behaviors, such as exploration.

### Adulthood: Female Urine-Induced USVs and Scent-Marking Behavior

Adult male mice exposed to females' urinary marks show a fast approach and prolonged sniffing behavior, emit ultrasonic vocalizations, a phenomenon known as the “ultrasonic courtship” vocalizations (Nyby, 1983; Holy and Guo, 2005; Arakawa et al., 2009; Wöhr and Schwarting, 2013), and deposit a large number of scent marks (Hurst, 1989; Arakawa et al., 2007), especially around the urinary source (Roullet et al., 2011). In this study, congenitally blind mice exhibited shorter latency to first sniff and higher duration sniffing the female urine spot, together with a shorter latency to emit the first female urine-induced USV call. The total number of calls, however, was similar between blind and sighted mice. Previous studies using the buried food test showed that blind rodents had a shorter latency to detect environmental odorants such as an appetent olfactory source than sighted congeners in congenitally blind ZRDBA mice (Touj et al., 2021) in visually deprived C57BL6 mice and rats (Zhou et al., 2017). To the best of our knowledge, our results highlight for the first time the importance of social olfactory cues (female urine) detection and their impact on acoustic behavior in anophthalmic male mice. In addition, histological and structural imaging studies reported larger olfactory bulbs and hypertrophy of brain areas involved in the olfactory processing (e.g., the anterior olfactory nucleus, the olfactory tubercle, the piriform cortex) in anophthalmic ZRDBA mice, supporting their heightened olfactory performance (Touj et al., 2020, 2021).

Accordingly, a longer time spent exploring the urinary source observed in blind mice may indicate a high attentional/motivational process toward odor stimuli. Similarly, increased attentional processes were found in our congenitally blinded mice in a two-odor choice test in response to positive odors (i.e., peanut butter and vanilla scent) compared to sighted mice (Touj et al., 2020). Thereby, visually impaired humans and non-human animals pay more attention to non-visual cues, processing them more efficiently compared to sighted individuals, which might enhance non-visual abilities (Kujala et al., 1997; Liotti et al., 1998; Hugdahl et al., 2004; Collignon et al., 2006; Collignon and De Volder, 2009; Ferdenzi et al., 2010; Beaulieu-Lefebvre et al., 2011; Pigeon and Marin-Lamellet, 2015; Topalidis et al., 2020).

Regarding the scent-marking behavior, the total surface marked was not different between sighted and blind adult mice in the whole cage and at 10 cm^2^ around the female urine drop. Dominant male mice tend to mark more than the subordinate ones (Desjardins et al., 1973; Rich and Hurst, 1998), so this result suggests that congenital blindness does not affect social ranking and reproductive-like behavior (both phenotypes are housed within the same cage). Further studies should examine in detail male social hierarchy in the context of sensory deficits such as visual deprivation. Finally, we reported a similar locomotor activity between the two groups in this test, which was also observed in Touj et al. (2020) when mice are assayed to an olfactory test.

Interestingly, in sighted, but not in the blind, adult male mice, there was a significant correlation between the number of female urine-induced calls and the total number of scent marks produced. This discrepancy may suggest an altered communication behavior in blind male mice in response to female urine, with a weaker coherence between the communication modalities (call emission and scent marking). This result is reminiscent of previous studies in a mouse model for autism, where the correlation between scent marking and USV emission appeared incoherent in the autism-like male group compared to the controls (Wöhr et al., 2011).

In conclusion, blind and sighted adult male mice exhibited differences in social communication and behavior in response to female urine stimuli.

### Longitudinal Correlation of Acoustic Communication With Other Behaviors

The longitudinal analysis of the acoustic and related social behaviors across the three developmental stages examined in this study, namely, early, juvenile, and adult stages, show that in sighted mice, but not in the blind, the number of USVs emitted during the first maternal isolation is correlated with the total number of scent marks in the adult. The calls emitted by the mice at PND 7 during the isolation might indicate a high level of arousal, attention, and motivation in the pups, which may then be reflected in and predict the scent-marking behavior in the adult.

It should be noted that both behaviors are crucial for the social life in the mouse, and this correlation may indicate a common biological foundation and neurological pathway (Demir et al., 2020). In contrast, social play behavior in the juvenile has been described as a unique category of behavior and does not predict future adult social, sexual, or aggressive behavior (Vanderschuren et al., 1997).

## Conclusion and Perspectives

This study investigated acoustic communication and associated behaviors in a mouse model of congenital blindness using a longitudinal approach. Early blind mice compared to sighted counterparts showed (1) no deficit in USVs' emission and social odor discrimination and preference at PND 7; (2) no difference in social play-induced USVs' emission but deficits in social behaviors (following and sniffing a congener) together with an increased exploratory behavior of the cage, without affecting the number of USV calls at PND 35; and (3) faster and longer exploration of female urine stimuli, faster emission to the first call, no differences in the number of calls and scent marks deposited in male adults. These findings indicate that congenital visual deprivation results in altered acoustic communication in the adult and modified social behaviors in juvenile and adult mice. Future studies should explore the qualitative analysis of the USVs emitted and examine the behavioral impact of playback calls, particularly within the mother-pup dyad, to understand with further details the foundation and development of acoustic communication of our mouse model of congenital blindness.

## Data Availability Statement

The raw data supporting the conclusions of this article will be made available by the authors, without undue reservation.

## Ethics Statement

The animal study was reviewed and approved by Animal Care Committee of the Université du Québec à Trois-Rivières, in accordance with the guidelines of the Canadian Council on Animal Care.

## Author Contributions

NB, EM-G, FIR, and SA: study conception and design. NB and SA: data collection. NB, JF, FIR, and SA: analysis and interpretation of results. NB and EM-G: draft manuscript preparation. All authors revised and approved the final version of this manuscript.

## Funding

This work was funded by the Natural Sciences and Engineering Research Council of Canada (NSERC) (2017-06942) and the MITACS Globalink Research Program.

## Conflict of Interest

The authors declare that the research was conducted in the absence of any commercial or financial relationships that could be construed as a potential conflict of interest.

## Publisher's Note

All claims expressed in this article are solely those of the authors and do not necessarily represent those of their affiliated organizations, or those of the publisher, the editors and the reviewers. Any product that may be evaluated in this article, or claim that may be made by its manufacturer, is not guaranteed or endorsed by the publisher.
